# Sequence homology in eukaryotes (SHOE): interactive visual tool for promoter analysis

**DOI:** 10.1186/s12864-018-5101-3

**Published:** 2018-09-27

**Authors:** Natalia Polouliakh, Paul Horton, Kazuhiro Shibanai, Kodai Takata, Vanessa Ludwig, Samik Ghosh, Hiroaki Kitano

**Affiliations:** 1 Sony Computer Science Laboratories Inc., 3-14-13 Higashigotanda, Shinagawa-ku, Tokyo, 141-0022 Japan; 2 Department of Ophthalmology and Visual Sciences, Yokohama City University, 3-9 Fukuura, Kanazawa-ku, Yokohama City, Yokohama, 236-0004 Japan; 3 Systems Biology Institute, 5-6-9 Shirokanedai, Minato-ku, Tokyo, 108-0071 Japan; 4 AIST, Artificial Intelligence Research Center, 2-4-7 Aomi, Koto-ku, Tokyo, 135-0064 Japan; 5 Department of Computer Science, Tokyo Institute of Technology, 2-12-1 Ookayama, Meguro-ku, Tokyo, 152-8552 Japan; 6 Department of Biology, ETH Zurich, Wolfgang-Pauli-Strasse 27, 8093 Zurich, Switzerland

**Keywords:** Comparative genomics, Transcription regulation, Gene network

## Abstract

**Background:**

Microarray and DNA-sequencing based technologies continue to produce enormous amounts of data on gene expression. This data has great potential to illuminate our understanding of biology and medicine, but the data alone is of limited value without computational tools to allow human investigators to visualize and interpret it in the context of their problem of interest.

**Results:**

We created a web server called SHOE that provides an interactive, visual presentation of the available evidence of transcriptional regulation and gene co-expression to facilitate its exploration and interpretation. SHOE predicts the likely transcription factor binding sites in orthologous promoters of humans, mice, and rats using the combined information of 1) transcription factor binding preferences (position-specific scoring matrix (PSSM) libraries such as Transfac32, Jaspar, HOCOMOCO, ChIP-seq, SELEX, PBM, and iPS-reprogramming factor), 2) evolutionary conservation of putative binding sites in orthologous promoters, and 3) co-expression tendencies of gene pairs based on 1,714 normal human cells selected from the Gene Expression Omnibus Database.

**Conclusion:**

SHOE enables users to explore potential interactions between transcription factors and target genes via multiple data views, discover transcription factor binding motifs on top of gene co-expression, and visualize genes as a network of gene and transcription factors on its native gadget GeneViz, the CellDesigner pathway analyzer, and the Reactome database to search the pathways involved. As we demonstrate here when using the CREB1 and Nf-κB datasets, SHOE can reliably identify experimentally verified interactions and predict plausible novel ones, yielding new biological insights into the gene regulatory mechanisms involved. SHOE comes with a manual describing how to run it on a local PC or via the Garuda platform (www.garuda-alliance.org), where it joins other popular gadgets such as the CellDesigner pathway analyzer and the Reactome database, as part of analysis workflows to meet the growing needs of molecular biologists and medical researchers. SHOE is available from the following URL http://ec2-54-150-223-65.ap-northeast-1.compute.amazonaws.com

A video demonstration of SHOE can be found here: https://www.youtube.com/watch?v=qARinNb9NtE

**Electronic supplementary material:**

The online version of this article (10.1186/s12864-018-5101-3) contains supplementary material, which is available to authorized users.

## Background

The analysis of gene regulatory regions is a centrally important problem in biology. Many experimental [1–3] and computational methods [4, 5] have been developed to address this problem. Despite these efforts and considerable progress, the analysis of eukaryotic gene regulatory regions remains difficult. One fundamental reason for this is that the binding sites of transcription factors (TFs) are only partially determined by their intrinsic sequence specificity; they are also strongly affected by factors including post-translational modification, interactions with other proteins, and the epigenetic state of the genome. Moreover, TF binding events are not necessarily all functional. Thus, effective promoter analysis is not just a simple matter of reporting motif matches or scores, but rather it demands careful consideration of multiple sources of supporting evidence such as the evolutionary conservation of potential binding sites and the coherence of the set of genes with promoters containing them regarding co-expression and cellular pathways.

Ideally a computer program could automatically and reliably combine all available evidence, but this has not yet been achieved. While many excellent motif discovery tools, such as CONSENSUS [6], the Gibbs sampler [7, 8], CRMD [9], and MEME [10], and alignment-based programs, such as rVista [11], ConSite [12, 13], Footer [12], and GPminer [14], have been developed, the current analytical demands require more than a simple output of putative TF binding sites. One example of an attempt to build an analysis workflow is PAINT [15] where regulatory analysis is represented in gene networks utilizing public software tools with original analysis.

SHOE takes a different approach; it tries to provide human biology experts with an interactive, visual presentation of the available evidence to facilitate the exploration and interpretation of transcription regulation analysis results. We demonstrate the SHOE analytical workflow by analyzing the CREB1 and Nf-κB datasets.

## Implementation

SHOE consists of a server application and front-end interface. The server application and its database run on Vagrant. Vagrant is virtualization software that enables the setting up of a software environment on any platform such as Windows or OS X. The server application is implemented in PHP 5, which is one of the most well-known Web application languages. The SHOE server connects and stores analysis data in a MySQL database. Some of the back-end programs are implemented in C language, Perl, and shell script. The client-side application is written in HTML and JavaScript.

GeneViz is a network visualization application created for SHOE. It shows multiple graphs of genes and transcription factors in one window. Users can compare common or different elements on these graphs and search for genes on them. GeneViz is written in JavaScript and is seamlessly integrated with SHOE, which can easily import data from it. SHOE also provides a gadget for the Garuda platform implemented in Java and installed as a plugin for CellDesigner (http://www.celldesigner.org). Garuda is an open platform that provides a framework to connect, discover, and navigate through different applications in the fields of biology and medicine [16]. SHOE can interact with CellDesigner and other applications on the Garuda platform. A manual explaining how to connect SHOE to CellDesigner is available on Garuda.

## Input and output

**Fig. 1:**
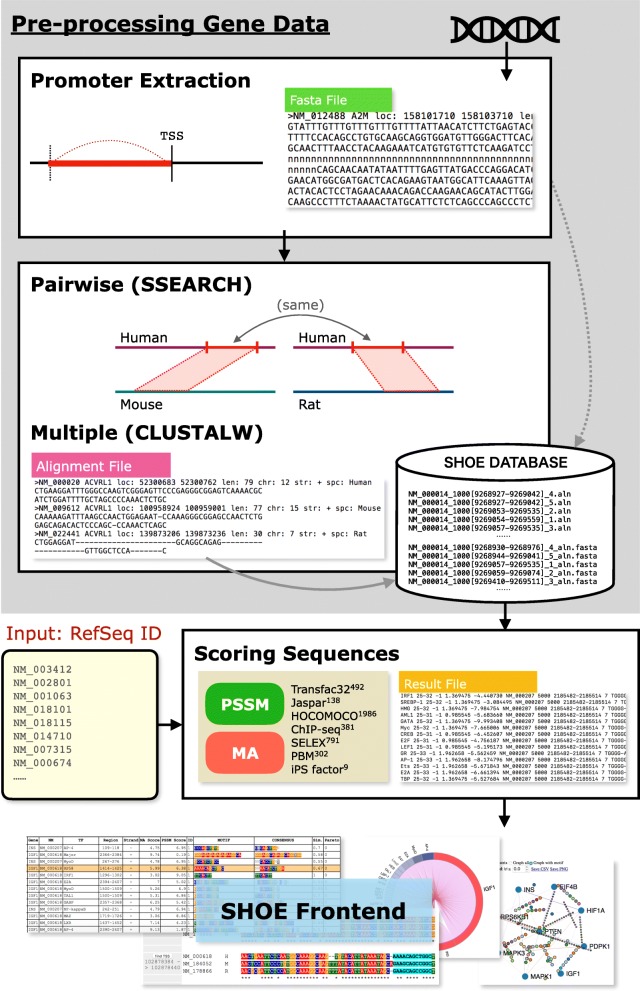
Flowchart of the execution of web-tool SHOE. Promoter extraction is followed by pairwise and multiple alignment of three species. Open source public matrices are matched to the human sequence if mouse and rat are aligned to human sequence region with similarity higher 50%. Finally, motifs with similarity score ≥ 0.5 are collected. Pearson correlation computes the co-regulation of genes in the dataset. (Detailed can be found in Algorithm section)

**Fig. 2:**
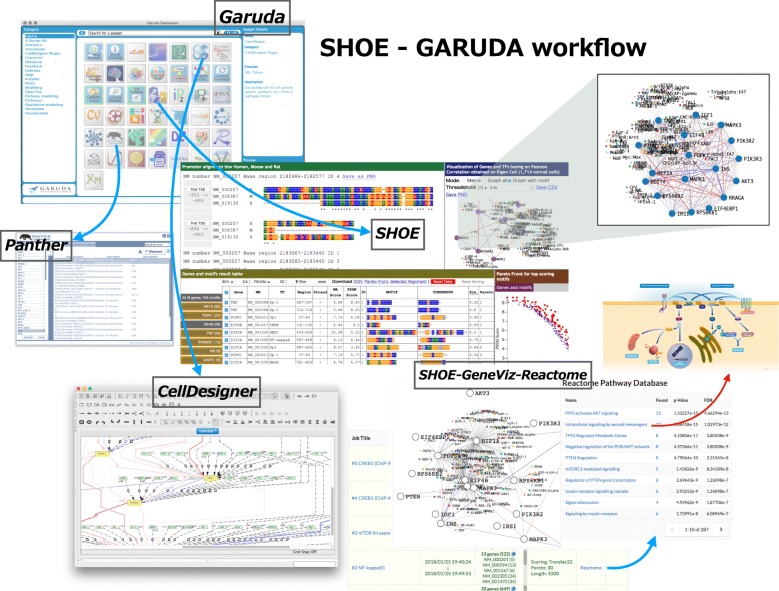
Example of SHOE-Garuda workflow. SHOE is a member of the Garuda platform. It can output results and acquire data from other tools, such as Panther, CellDesigner, and others via gadget connecting SHOE and Garuda platform. To CellDesigner SHOE connects with and without Garuda gadget, using a solely CellDesigner plugin. SHOE has its native gadget GeneViz for the visualization of the gene network obtained on Pearson correlation analysis. GeneViz does a straightforward search in the Reactome database to visualize pathways present in the analysis dataset

As input, SHOE takes a list of human, mouse, or rat Refseq gene ids. First, SHOE assesses if the received gene has orthologs in the other two species. If so, SHOE extracts the promoter and undertakes the computational steps depicted in Fig. 1 and all steps are descibed in the following Algorithm part. As output, SHOE returns a list of genes with motif hits that are common among the three species with the SHOE criteria and gene co-expression. Users can view this output in several ways, as shown in Fig. 2. A motif hit table and gene network window are provided with options that can help users to select the part of the data of their interest, sort by scores, change cutoffs, and save data in text and graphical formats. In order to obtain information on the transcription factor binding, ArrayExpess [17] ChIP-seq data on Liver has been added to the results table. By clicking on the motif in the table User can judge how close the peak location to the motif identified. For further addition of tissue-specific data upon users request the workflow is provided in SHOE Manual “Adding ChIP-seq Array Express tissue-specific data” page. The user can change the expression interaction threshold, and these changes will be reflected in the results table and saved. All tables can be reset. SHOE returns a gene co-expression network. Pareto front selection allows the user to stipulate how strongly evolutionary conservation should be weighted vis-à-vis PSSM scores. Emphasizing the former might be suitable for those who are looking for novel motifs in well-conserved alignment blocks, while the dominance of the latter will be more interesting for clinicians working on human data.

## Algorithm

A flowchart of the execution of the web-tool SHOE algorithm is shown in Fig. 1.

An ortholog gene list of 6,669 human, mouse, and rat genes was obtained from the DBTSS database [18] of experimentally verified transcriptional start sites on the level of mRNA expression.

### Local pairwise and multiple local alignments

Each human promoter was aligned with his/her orthologous partners from the list of orthologs (mouse and rat) by using the SEARCH local alignment program [19]; the execution of this program was repeated for promoters defined with length 1000, 2000 and 5000 nt, respectively. In the cases where the same human promoter region was aligned to mouse and rat promoters with a similarity higher than 50%, the three genomes’ respective regions were extracted, realigned with ClustalW [20], and finally stored in a MySQL database to avoid recomputing.

### Multiple alignment (MA) score

To evaluate the degree to which an observed region in an alignment of the three species should contribute to our belief that it is part of a conserved region, we adopted an estimate of the likelihood ratio of observing the region in an alignment of orthologous promoters versus that of observing the region in an alignment of unrelated promoters.

To obtain this estimate, first, we randomly selected 1,000 orthologous triplets (human, mouse, and rat genes) and repeatedly aligned their orthologous promoters of length 5,000 nt. After 835 orthologous three-species alignments (238,800 bp total length) were obtained with ClustalW, the frequency of each alignment column was observed and stored in an orthologous alignment frequency table, i.e., an *ortho* table. In the same manner, 1,000 non-orthologous triples were randomly selected, and their promoters of length 5,000 nt were repeatedly aligned. After 1,260 non-orthologous three-species alignments (239,600 bp total length) were obtained with ClustalW, the frequency of each possible alignment column was observed and stored as a *random* alignment frequency in a table.

We refer to the obtained alignment column frequencies as the “ortho” alignment and “random” alignment frequencies, respectively. Using those column frequency tables, we define the multiple alignment (MA) scores using the following formula:

1 $$ MAscore={\log}_{10}\frac{\prod \limits_m\Pr \left(c\left| ortho\_ alignment\right.\right)}{\prod \limits_m\Pr \left(c\left| random\_ alignment\right.\right)}, $$

where *c* is the probability of the observed pattern in each column (calculated using pattern frequencies from orthoalignment and *random* alignment tables), and *m* is the length of the alignment region, which for our application is equal to the motif length *m*.

### Position-specific scoring matrix (PSSM) score

After multiple alignment scores have been calculated for the human-mouse-rat regions of length *m* to evaluate the degree of similarity between the identified conserved sequence and known transcription factor binding sites, SHOE uses its motif-scoring module, which calculates similarity scores with matrices in public databases such as Transfac32 (492 matrices) [21], Jaspar (138) [22], HOCOMOCO (1,986) [23], ChIP-seq (381) [2], SELEX (791) [1], PBM (302) [24], and iPS factor matrices (9) [25], which was created by the authors and is described in the SHOE manual page.

The PSSM-related score is calculated using the following formula:

2 $$ PSSMscore=\sum \limits_{i=1}^m{\log}_2\frac{count_{x_i}+{pseudocount}_{x_i}}{\sum \limits_{x=\left\{A,T,G,C\right\}}{count}_{x_i}+\sum \limits_{x=\left\{A,T,G,C\right\}}{pseudocount}_{x_i}}, $$

where *pseudocount* = 1, and *m* is the motif length.

To represent the scores as positive values, the PSSM score is subtracted from 10.

### Pareto-optimal front for motif selection

Since SHOE identified putative TF binding sites via two sources of information (multiple alignment scores and PSSM scores), we decided to apply the Pareto-optimal front method to optimize this multi-objective solution [26]. The problem of exploring solutions under multiple objectives has traditionally been tackled in engineering, utilizing a so-called “desirability function”, whose value is 1 when the response takes values considered valid by the analyst and 0 otherwise. The Pareto number evaluates the degree to which both the PSSM score and the MA score are favorable. The application of the Pareto front in SHOE is shown in Additional file 1: Figure S1.

The Pareto number of (x_i_,y_i_), where x_i_ is the MA score and y_i_ is the PSSM score, is calculated by

3 $$ Pareto\left({x}_i,{y}_i\right)=\#\left\{x\in MA,y\in PSSM,{x}_i<x\wedge {y}_i<y\right\}+1, $$

where MA and PSSM are the sets of scores.

### Motif enrichment score (MES)

To compare the frequencies of motifs in two user datasets, we acquired the motif enrichment score (MES) [27], which is applied to the standard deviation of a binomial distribution and is a measure of the evolutionary conservation of motifs. Here, we use that metric in a different context as a sample size-dependent measure of the enrichment of matches to a motif in a gene set. In our application, the MES of a motif m is given by

4 $$ MES=\frac{K-{Np}_0}{\sqrt{Np_0\left(1-{p}_0\right)}}, $$

where K is the number of genes with a match to motif m in the gene set of interest, N is the total number of genes in the gene set, and p0 is the frequency of the same motif in a background set of genes.

### Computing gene co-regulation on Eigen cell

Considering that genes with similar transcriptional profiles might be affected by the same transcriptional mechanism, we calculated the Pearson correlation coefficients for all gene pairs in the datasets based on 112 “Eigen cell” synthetic expression profiles available from the Cell Montage web site (http://cellmontage.cbrc.jp/) [28].

For the reader’s convenience, here we briefly outline whom those Eigen cells were computed for. First, 5000 human cell profiles from the Gene Expression Omnibus database [29] were classified into 1,714 normal cell profiles consisting of 89 cell or tissue types. Then, a standard principal component analysis was conducted to reduce the number of dimensions. The 1,714 profiles were transformed by eigenvectors and reduced to the top 112 informative “Eigen cells” using the Keiser-Guttman criteria [30]. The reduced “highly informative gene expression data” or “Eigen cell profiles” as well as the raw cell data are freely available on the Cell Montage website and are adopted in SHOE for calculation of co-expression of genes in the gene set received by SHOE as an input.

## Results

**Fig. 3:**
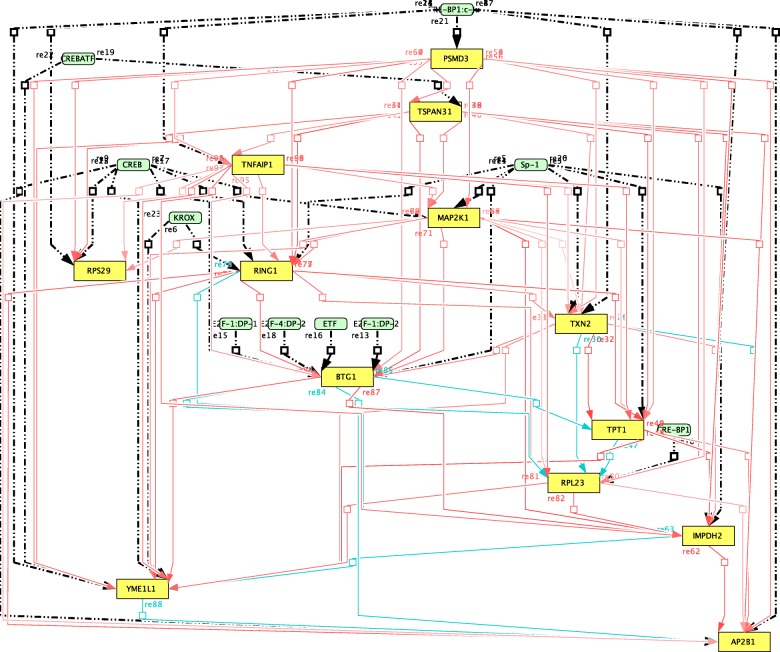
CellDesigner Map shows the SHOE predicted genes and transcription factors of the CREB1 regulated genes visualized in the CellDesigner pathway editor. Blue lines correspond to experimentally verified interactions from the GeneMANIA database. Genes are positively co-regulated and connected with red lines

**Fig. 4:**
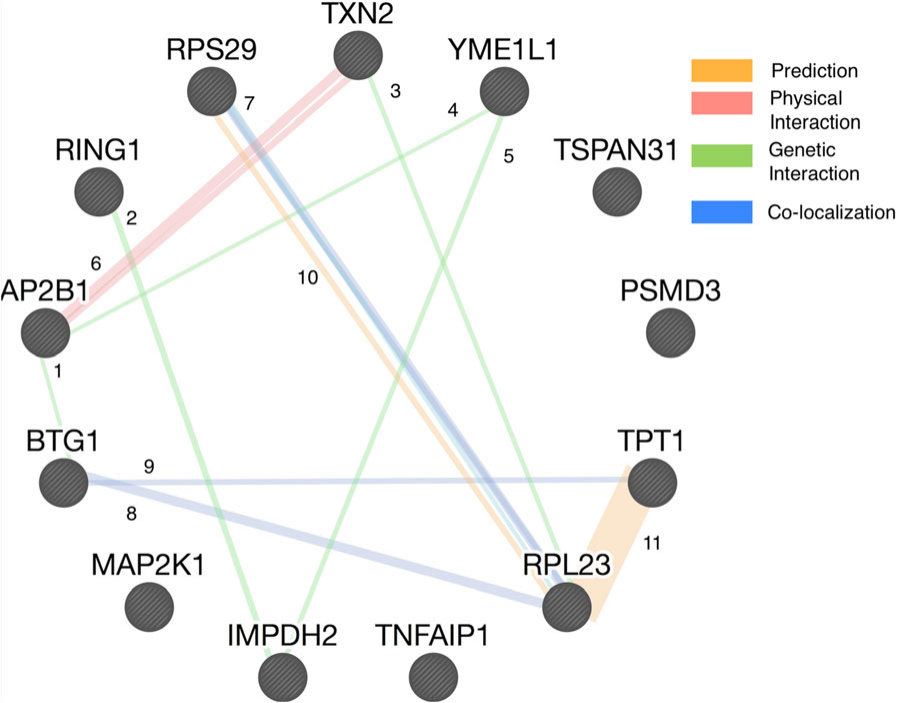
SHOE-predicted CREB1 regulated genes visualized in GeneMANIA database. Numbers correspond to SHOE-predicted interactions in Fig. 3

**Fig. 5:**
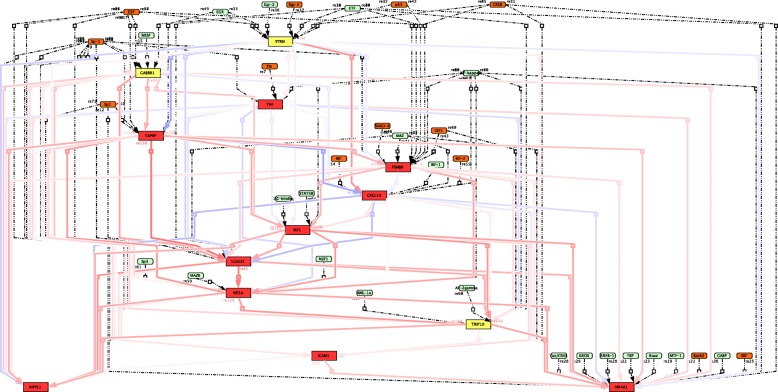
CellDesigner Map shows the SHOE predicted genes and transcription factors of the Nf-κB-regulated network. Interactions between transcription factors (orange; literary evidence for interaction with Nf-κB, green; no evidence for interaction with Nf-κB) and genes (red; literary evidence of Nf-κB regulation, yellow; no evidence of Nf-κB regulation) are depicted with dashed lines. The co-regulations between genes are shown; positive (red lines) and negative (blue lines)

To test SHOE, from ChIP Atlas (http://chip-atlas.org) we selected 1000 human promoters (1000 nt length) as the target genes of the CREB1 transcription factor and 1000 human promoters (1000 nt length) as the target genes of the Nf-κB1 transcription factor. With predicted target genes, gene-interaction maps have been visualized in CellDesigner (Figs. 3, 4 and 5). The following two paragraphs give a biological discussion of the results of SHOE analysis of CREB1 and Nf-κB1.

### CREB1 dataset

For the 1000 target genes tested with CREB1, 37 had orthologs in both mouse and rat genes. Additional file 2: Figure S2 shows the changes in the number of genes in the dataset depending on the similarity threshold with the consensus sequence. For the visualization, we applied a strict selection criteria, selecting only 13 genes with a similarity PSSM consensus score of ≥0.75 and a Pearson correlation score of |r| ≥ 0.6, as shown in Additional file 2: Figure S2. Those 13 genes predicted as CREB1 targets are visualized in the CellDesigner [31] map through the original SHOE-CellDesigner plugin, as shown in Fig. 3, and compared to the known interactions in GeneMANIA [32], as shown in Fig. 4. By comparing our CREB map to GeneMANIA data, we found that several SHOE predicted interactions have already been discovered and experimentally verified.

#### Verified gene/protein experiment-based interactions between SHOE and GeneMANIA

The blue lines on the CellDesigner map in Fig. 3 and GeneMANIA in Fig. 4 point to the interactions based on co-retention frequencies using radiation hybrid genotyping data [33]. Due to the fact that many of the potential interactions identified by SHOE are known, it is highly likely that some of the remaining predicted interactions are genuine novel ones. Another example is the protein interaction between AP2B1 and TXN2 (Figs. 3 and 4, interaction no. 6, respectively) that was found when screening all pairwise combinations of open reading frames from human ORFeome v 5.1 generated by a binary protein-protein interaction map [34]. As AP2B1 is part of intracellular transports and TXN2 plays a role in the control of mitochondrial reactive oxygen species homeostasis and apoptosis regulation according to the DAVID database [35], the two proteins could interact in multiple pathways.

#### Verified experiment-based co-localization between SHOE and GeneMANIA

The co-localization of BTG1 and RPL23 (Figs. 3 and 4, interaction No. 8) was identified when microarray data was combined with extensive genome annotations [36]. The co-localization of BTG1 and TPT1 (Figs. 3 and 4, interaction no. 9, respectively) was found when microarrays were used to monitor splicing, providing experimental evidence for alternative splicing events [37]. As TPT1 is a regulator of cellular growth and proliferation, BTG1 is a regulator of the cell cycle in the DAVID database, and they are co-localized, it is likely that these genes will genuinely interact. The prediction of an interaction between TPT1 and RPL23 (Figs. 3 and 4, interaction no. 11, respectively) was found by constructing a functional protein network by extending curated pathways with non-curated sources [38].

#### Verified predictions between SHOE and GeneMANIA

Some of the genes identified such as mitogen-activated protein kinase kinase 1 (MAP2K1), ribosomal protein L23 (RPL23), and ribosomal protein S29 (RPS29) are part of the conserved structures or pathways in the DAVID database. RPL23 and RPS29 (Figs. 3 and 4, interaction no. 7, respectively) are ribosomal proteins that take part in nuclear-transcribed mRNA catabolic processes essential to the survival of cells. MAP2K1 is part of multiple signaling pathways and many pathways in cancer. Identifying MAP2K1 as a potential target of CREB is a step closer to uncovering its regulation, network, and what role it might play in different cancer types. CREB was identified as part of the transcriptional regulation of antioxidant enzymes in brain tissue and as a regulator so that the animal responds properly to stressful conditions [39]. Several of the genes SHOE identified such as MAP2K1 and TXN2 are listed on DAVID as part of a stress response. TXN2, which is an oxidoreductase, was identified as being a potential CREB target gene [40]. This further supports our predictions and strengthens the credibility of SHOE as a predictor of interactions between genes.

### Nf-κB dataset

Out of the 1000 genes tested for Nf-κB, SHOE identified 17 genes containing conserved Nf-κB binding domains with a similarity score in the PSSM consensus of ≥0.5. Thirteen genes depicted in the CellDesigner Nf-κB map, where genes whose ratio of similarity scores to consensus sequence is higher than 0.6, are visualized in Fig. 5. Ten genes are confirmed by the literature to be target genes of Nf-κB (Fig. 5, red rectangles), making the other three the predicted new candidates (Fig. 5, yellow rectangles).

#### Apoptosis

It is known that transcription of tumor necrosis factor (TNF), which is involved in apoptosis, is induced by Nf-κB in mice [41]. HIF1A included in our map demonstrates that Nf-κB is a direct modulator of HIF1A expression [42]. The literature reported that it is possible for NR4A1 (Nur77) to assist Nf-κB binding to promoters of anti-apoptotic genes [43]. Another apoptotic gene in our network is LGALS1 (galectin-1) whose expression is controlled by the Nf-κB signaling pathway, and the LGALS1 gene is a direct target of the Nf-κB p50 subunit [44].

#### Immune system and insulin

Many Nf-κB target genes are part of the immune system such as the ICAM1 gene. qPCR analysis identified ICAM1 as a target of Nf-κB [45], and Nf-κB activation was shown to induce ICAM1 expression [46]. PSMB9 (LMP2) was found to be coordinately regulated by using an Sp1-GC box and Nf-κB site [47]. Sp-1 itself is predicted by SHOE to target PSMB9. Nf-κB is found to be essential for the basal activity of the mouse TAPBP (tapasin) promoter [48]. Furthermore, in neuroblastoma cells, Nf-κB was found to synergize with IRF1 in enhancing tapasin [48]. The IRF1 regulatory region itself was found to bind Nf-κB [49]. Published experimental results demonstrate that in colonic epithelial cells, using the Nf-κB pathway, IL-1β may induce CXCL10 [50]. INPPL1 (SHIP2) is involved in the regulation of insulin and also plays a role in actin remodeling [51]. It is known that palmitate could induce SHIP2 expression in skeletal muscle via the activation of Nf-κB pathways [51].

#### Known and new transcription factors predicted by SHOE

SHOE predicted another three genes that have not yet been associated with Nf-κB in mice and rats but have been predicted by SHOE: TRIP10, STRN, and GABBR1. Also, several transcription factor candidates were found to potentially co-regulate these genes together with Nf-κB.

TRIP10 (CIP4), which is involved in insulin signaling and actin reorganization, has been identified as a potential target gene of Nf-κB. TRIP10 (CIP4) is required for the translocation of GLUT4 to the plasma membrane (http://www.uniprot.org).

SHOE predicted that LEF1 and Sp1 among other transcription factors affect TRIP10 expression. Nf-κB was found to regulate the LEF1 transcription factor and interact with the Sp1 transcription factor [52]. The above result supports the idea that Nf-kB co-regulates with TRIP10, LEF1, and Sp1. Since both TRIP10 and INPPL1 are part of insulin signaling and actin remodeling and are predicted to be regulated by Nf-kB, they might be involved in the insulin-signaling pathway together.

STRN, which is a calmodulin-binding protein, and GABBR1, which is a receptor for gamma-aminobutyric acid, are also predicted by SHOE to have Nf-kB binding sites in their promoters. Interestingly, Nf-kB is reported to be a constitutive transcription factor in glutamatergic neurons [53]. Thus, STRN and GABBR1, as part of an insulin pathway, may be involved in learning and memory.

We report that CREB, Egr-3, E2F, and p53 regulate STRN, and accordingly, Nf-κB was found to regulate CREB [54]. Egr-3 [55] and E2F1, [56] both separately interact with Nf-kB. P53 and Nf-kB were found to synergistically upregulate multiple genes. Sp1 and E2F bind to the GABBR1 promoter, and these two transcription factors interact with Nf-kB.

Bach2 [57], IRF family members [58–60], SP3 [61], SRF [62], ETS proteins [63], SMAD4, and TGF-β [64] transcription factors are also known to interact with Nf-kB, which give us a chance to extend the present biological insights into Nf-kB activity. Out of 33 predicted transcription factors, 13 had been confirmed in combinatorial interactions with Nf-κB (Fig. 5, orange), supporting the possibility of Nf-κB co-regulating these genes.

### Exploration ChIP-seq peaks with SHOE

In order to provide Users with the insight of how the results of SHOE are overlapping with experimentally predicted transcription factor binding sites, we incorporated ChIP-seq Liver enhancer data into SHOE. With this amendment whichever geneset is analyzed the results can be viewed in overlap with ChiP-seq peaks in Liver, or other tissues by User request. On SHOE Manual page we put the detailed protocol of how to add ChIP-seq data from Array Express to SHOE. Thus all dataset we/users analyzed could be investigated on the presence of overlap with ChiP-seq peaks. As an additional example, we brought the dataset of 154 genes overexpressed in type 2 Diabetic Mouse Liver and present in SHOE orthologous list [65]. SHOE 24 identified genes of 154 as having cross-species conserved regions (Liver_ChIP-seq dataset in “Query List”). Several genes such as FIF4B, PKLR, IGFBP4, ATP5D shown overlap with ChIP-seq peaks (Additional file 3: Figure S3a). In the mTOR pathway dataset ChiP-seq locations have been found for two genes TNF and EIF4B, and in the promoter of TNF gene two peak locations were correctly predicted (Additional file 3: Figure S3b). Other genes in the mTOR pathway dataset did not have peak information. The above means that the analysis by SHOE might bring helpful information for the identification of possible transcription factor candidates within ChIP-seq regions of binding peaks.

To capture more Chip-seq peaks several considerations should be included into the methodology: a) since enhancer regions are less conserved comparing to promoters, they can have weaker scores in multiple sequence alignment, thus being left out, as in the case of ATP5D gene, when both sides of peak are very well conserved but peak itself is missed because of weaker multiple alignment similarity; b) motifs in peaks are more corrupted/(having more mismatches) with consensus sequence of known binding site, as it is observed by SHOE (similarity scores to consensus around 0.5, thus the cutoff threshold should be set significantly lower for ChIP-seq peaks analysis); c) some peaks are distantly allocated from the TSS (to more than 10,000 bp), which means that more sensitive, i.e. partial sequence search directly on those regions might be more appropriate. Despite the above considerations are not included in current SHOE methodology, SHOE is successfully finding ChiP-seq locations in a range of cases thus increasing the confidence of the results that will augment the interest of the user to the software.

## Conclusions

Through case studies we demonstrate the utility of SHOE in visualizing and exploring potential regulatory interactions involving TFs. The analysis made by SHOE further emphasizes the evolutionary importance of CREB in stress-regulated responses and the regulation of antioxidant enzymes. Moreover, for the example of Nf-kB, 10 out of 13 predicted target genes had evidence in the literature supporting the predictions. Not only known but also novel potential target genes in the insulin pathway and the nervous system were identified, which may give insight into new pathways and how Nf-kB is involved in their regulation. For 11 out of 33 transcription factors, there were studies showing interactions with Nf-kB, portraying SHOE as a strong predictor of not only target genes but also potential transcription factors that might co-regulate target genes. Analysis on the MAPK pathway using the SHOE method have been discussed in a previous study [66]. By identifying the roles of genes and their relations to other genes, SHOE can be used to help create a potential network of gene interactions.

SHOE is connected to such tools as the CellDesigner pathway editor and analyzer [31], Percellome database [67], and Reactome database [68] via the Garuda platform and uses its native visualizer GeneViz to represent/compare several networks at once. As future work, shortly we plan to add other tools to join the SHOE analytic workflow to meet the growing needs of molecular biologists and medical researchers.

We also are considering adding the option to consider tissue-specific data such as ChIP-seq binding data and epigenetic data such as DNA methylation and histone modification.

## Availability and requirements

Project name: SHOE: Interactive visual tool for promoter analysis.

Project home page: http://ec2-54-150-223-65.ap-northeast-1.compute.amazonaws.com

Project demo: https://www.youtube.com/watch?v=qARinNb9NtE

Operating system(s): Windows / macOS / Linux.

Programming language(s): C, Perl, PHP, JavaScript.

Other requirements: Vagrant, VirtualBox.

Restrictions for use by non-academics: None.

## Additional files

Additional file 1: **Figure S1.** Pareto front score optimization on SHOE. A) Illustration of the conflict between two scores (MA score and PSSM score) and the concept of dominance; B) Visualization of several groups of scores using Pentachlorophenol response dataset (PCP), PCP_raw (red) denotes random score zone, PCP_ma (purple) denotes zone where MA score is high, PCP_pssm (blue) is the zone where the PSSM score is high, and PCPtrimmedLog (green) is the zone where a trade-off between two scores is taken. C) Example of the analysis with 30 top Pareto fronts with each type of motif shown in different shape and color. (PDF 360 kb)

Additional file 2: **Figure S2.** Trade-off on the number of genes in the dataset basing of motif similarity threshold to the consensus when Pearson correlation thresholds |r| ≥ 0.0 and |r| ≥ 0.6 are applied. (PDF 20 kb)

Additional file 3: **Figure S3.** Visualization of ChiP-seq peaks from ArrayExperss database idenyified in SHOE predictions. A) Demosntrates two genes from overexpressed in mouse liver in Diabet 2 condition; B) Demonstrate TNF genes of mTOR human pathway in which promoter two peaks according ChIP-seq analysis have been identified. (ZIP 499 kb)
