# Supplementary figures and images for: Sequence homology in eukaryotes (SHOE): interactive visual tool for promoter analysis
Source: BMC Genomics. 2018 Sep 27;19:715. doi: 10.1186/s12864-018-5101-3 (PMC6161448; doi:10.1186/s12864-018-5101-3)

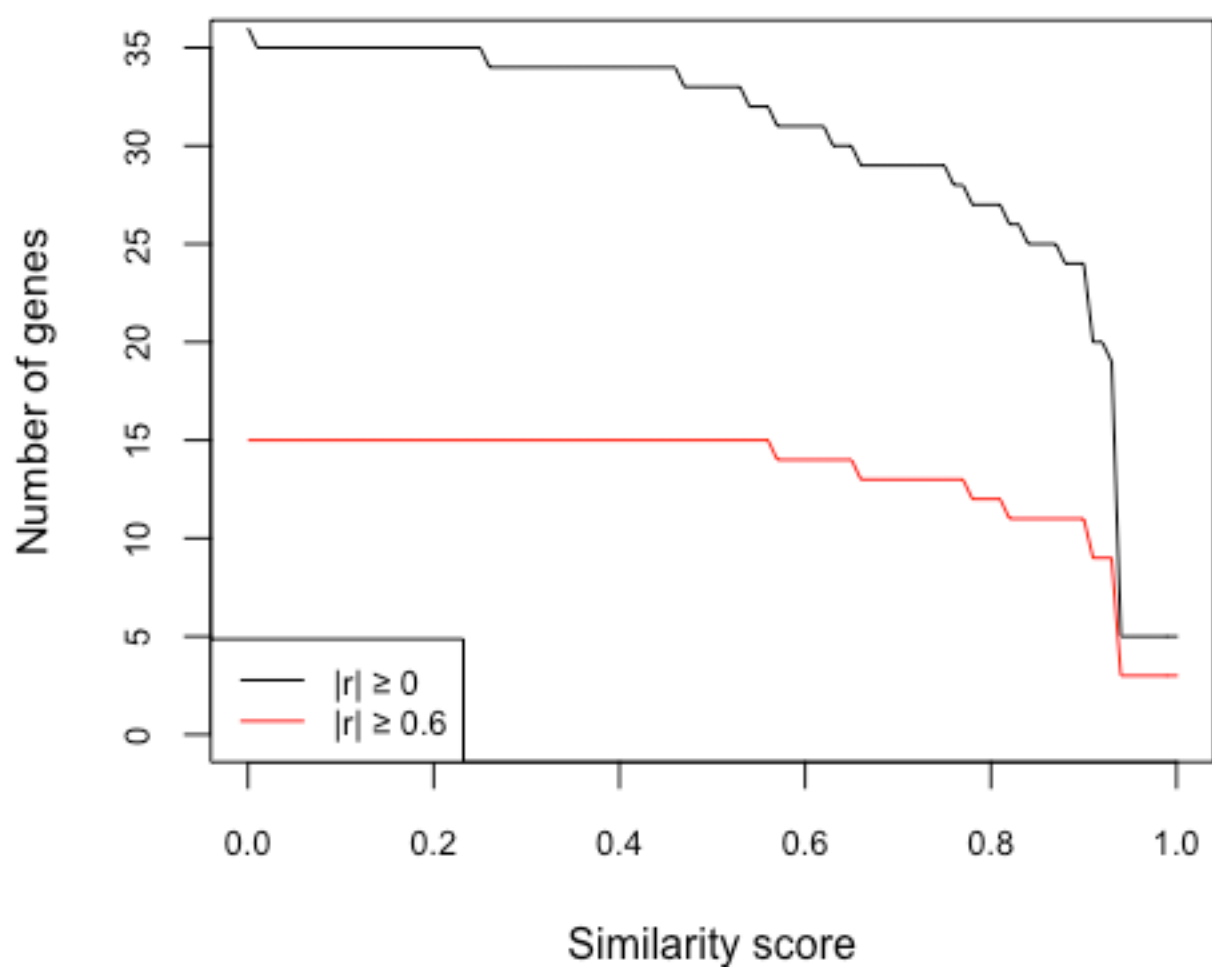

Supplement: Supplementary file 2 — Figure S2. Trade-off on the number of genes in the dataset basing of motif similarity threshold to the consensus when Pearson correlation thresholds |r| ≥ 0.0 and |r| ≥ 0.6 are applied. (PDF 20 kb) [file 12864_2018_5101_MOESM2_ESM.pdf]
