# Supplementary material for: Sequence homology in eukaryotes (SHOE): interactive visual tool for promoter analysis
Source: BMC Genomics. 2018 Sep 27;19:715. doi: 10.1186/s12864-018-5101-3 (PMC6161448; doi:10.1186/s12864-018-5101-3)
Supplement: Supplementary file 3 — Figure S3. Visualization of ChiP-seq peaks from ArrayExperss database idenyified in SHOE predictions. A) Demosntrates two genes from overexpressed in mouse liver in Diabet 2 condition; B) Demonstrate TNF genes of mTOR human pathway in which promoter two peaks according ChIP-seq analysis have been identified. (ZIP 499 kb) [file 12864_2018_5101_MOESM3_ESM.zip › Supplementary Figure 3a.pdf]

Diabet Type 2 genes in Liver: EIF4B and PKLR

a

Peak area (-136 to -123)

```
find TSS   NM_001417     H   AGAGTAAGC-----AGAAGCAGCA-GCGAAGCCCCGCCCTTCCCGCCACTCTTCCGGCAG
-162 ->    NM_145625     M   CCACTCTGTTCCATAGAATCAGCAAGTGCGACCCCGCCCTTCGCGCCGATCCAGCACCCA
-109       NM_001008324  R   CCACTCGGTTCCATAGAATCAGCAAGTGCGGCCCCGCCCTTCGCGCCTACCCAGCACCCA
                               *  *   *      ****  *****  *  *   ***********  ****    *    *
```

GeneID	Refseq	TF	strand	MA score	PSSM score		MOTIF	CONSENSUS	SIM	Pareto	human +/-	human dist	human width	human score
EIF4B	NM_001417	CREB	+	2.43	9.2	61	CGTCAG	CGTCAN	0.83	24	+	-84 -252	168	15.2
EIF4B	NM_001417	KROX	+	7.44	4.03	1	CCCGCCCTTCCCGC	CCCGCCCCCRCCCC	0.71	21	+	-84 -252	168	15.2
EIF4B	NM_001417	CREB	+	2.94	9.2	1	CGTCAG	CGTCAN	[illegible]	[illegible]	[illegible]	[illegible]	[illegible]	15.2
EIF4B	NM_001417	Sp-1	+	4.71	8.34	1	CCCCGCCCCT	CCCCGCCCCN	[illegible]	[illegible]	[illegible]	[illegible]	[illegible]	15.2
EIF4B	NM_001417	Sp-1	+	5.47	7.75	1	CCCCGCCCTT	CCCCGCCCCN	[illegible]	[illegible]	[illegible]	[illegible]	[illegible]	15.2
CHI3L1	NM_001276	CRE-BP1	+	1.41	9.57	5	TTACGTAA	TTACGTAA	1	11	-	-5183 -5559	376	201.8

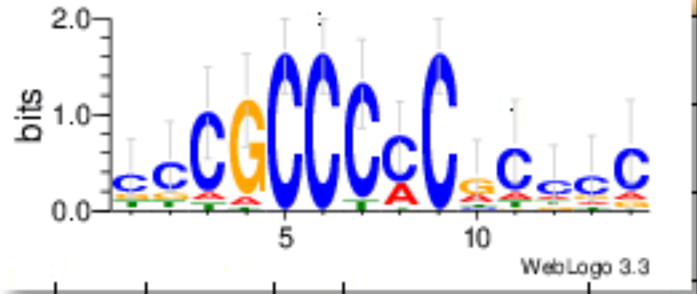


```
find TSS   NM_000298  H   CTCACCCTCTCCATCTCATCTCCTATGGGTGCCACTCT-------CCAAACCCTACAGAC
-3615 ->   NM_013631  M   ------CTTCTCACCCAGTCTCCCATGGGCGCTATTCTCCACTGGCCAAACTCTGTAGAA
-3563      NM_012624  R   ---------CTCACCCAGTCTCCCACGGGTGCTATTCCCCACTGACCAAACTCTGTGGAA
                                    ** *      *****  *  ***  **  *  **         ******  **    **
```

Peak area (-3547 to -3542)

```
find TSS   NM_000298  H   GCCACTGTGCAAAGCCGTCAGCCTCCTGGCCCCACAGCAGAGTGGAAAAGG
-3562 ->   NM_013631  M   GCCA-AGGACAAAGCCTTTGGC-TCCTTGCCCCAAATCAGAG---------
-3512      NM_012624  R   GCCCTGGGACAAAGCCTTTGGC-TCCTTGCCCCAAATCAGAGAAGAAGGGG
                          ***     *  *******  *   **  ****  ******  *  *****
```

gene	Refseq	TF	strand	MA score	PSSM score		MOTIF	CONSENSUS	SIM	Pareto	human +/-	human dist	human width	human score
PKLR	NM_000298	MAZ	+	3.09	8.86	1	GGGGAGGG	GGGGAGGG	1	31	-	-3218 -3578	360	354.6
PKLR	NM_000298	LEF1	+	1.6	9.26	6	TCAAAG	TCAAAG	1	29	-	-3218 -3578	360	354.6
PKLR	NM_000298	CREB	+	2.32	9.2	27	CGTCAG	CGTCAN	0.83	29	-	-3218 -3578	360	354.6
PKLR	NM_000298	AR	+	7.12	4.64	1	GGGACAGGGTGGCCT	GGTACANNNRTGTTCT	0.63	24	-	-3218 -3578	360	354.6
PKLR	NM_000298	LEF1	+	2.12	9.26	5	TCAAAG	TCAAAG	1	20	-	-3218 -3578	360	354.6
PKLR	NM_000298	Roaz	-	6.41	5.65	27	TCTCCTATGGGTGC	GCACCCAWGGGTGM	0.71	16	-	-3218 -3578	360	354.6
[illegible]	[illegible]	[illegible]	[illegible]	[illegible]	[illegible]	[illegible]	[illegible]	[illegible]	[illegible]	[illegible]	[illegible]	[illegible]	[illegible]	[illegible]

mTOR pathway : TNF gene

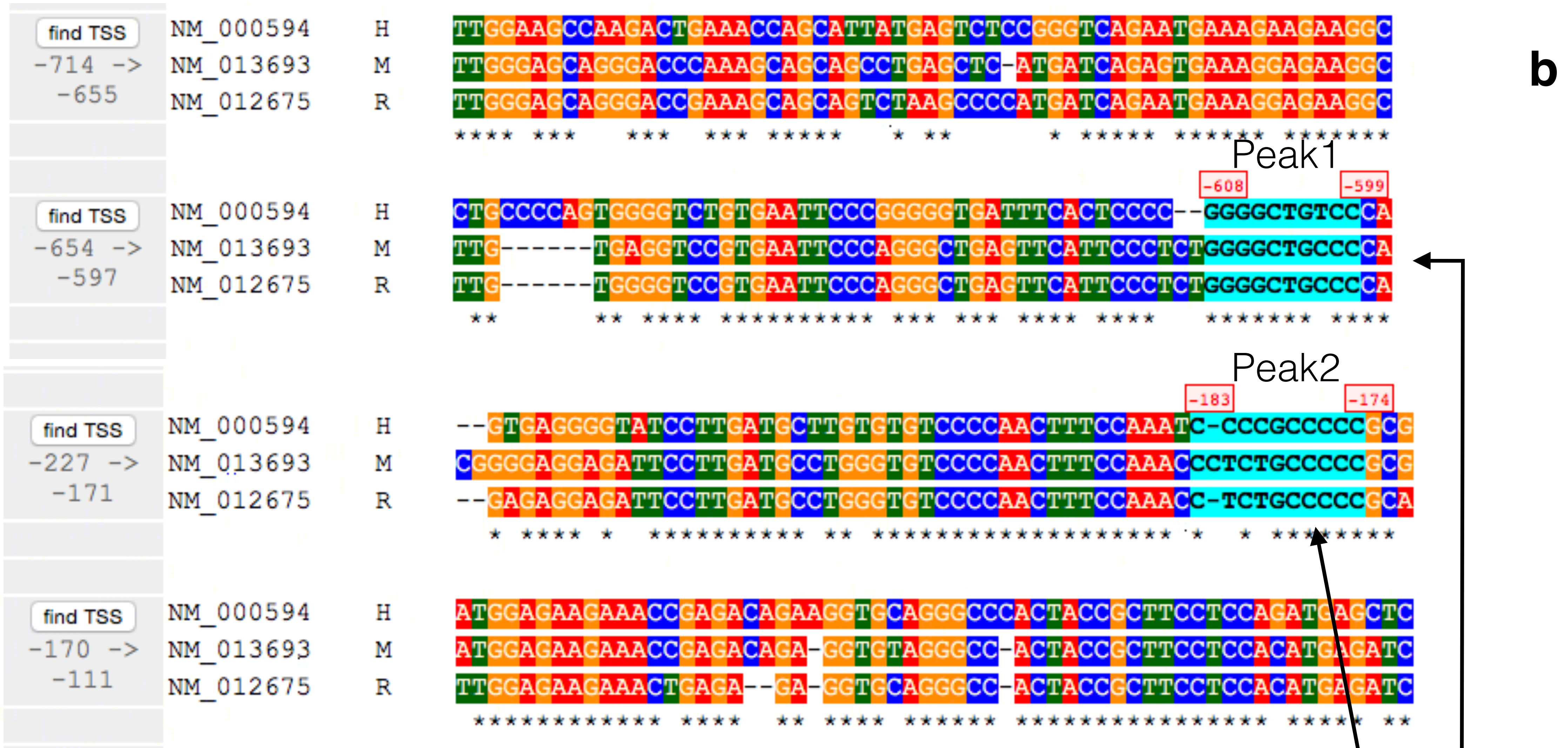

	gene	Refseq	TF	strand	MA score	PSSM score		MOTIF	CONSENSUS	SIM	Pareto	human<br>+/-	human<br>dist	human<br>width	human<br>score
☑	TNF	NM_000594	c-Rel	+	5.39	6.62	1	GGGGCTGTCC	SGGRNTTTCC	0.7	19	+<br>+<br>+	-501 -761<br>-174 -564<br>-868 -1449	260<br>390<br>581	97.9<br>211.4<br>351.8
☑	TNF	NM_000594	Sp-1	+	5.68	8.05	1	CCCCGCCCTC	CCCCGCCCCN	0.8	1	+<br>+<br>+	-501 -761<br>-174 -564<br>-868 -1449	260<br>390<br>581	97.9<br>211.4<br>351.8
☑	TNF	NM_000594	Sp-1	+	4.88	8.64	1	C-CCCGCCCCC	CCCCGCCCCN	0.55	1	+<br>+<br>+	-501 -761<br>-174 -564<br>-868 -1449	260<br>390<br>581	97.9<br>211.4<br>351.8
☑	TNF	NM_000594	STAT3	+	8.97	0.69	1	TGTGAATTCCCGGGGGTGATT	NGNNATTTCCSGGAARTGNNN	0.52	13	+<br>+<br>+	-501 -761<br>-174 -564<br>-868 -1449	260<br>390<br>581	97.9<br>211.4<br>351.8
☑	TNF	NM_000594	STAT1	+	3.82	8.05	1	GAATTCCC	CANTTCCS	0.69	15	+<br>+<br>+	-501 -761<br>-174 -564<br>-868 -1449	260<br>390<br>581	97.9<br>211.4<br>351.8
☑	TNF	NM_000594	PU.1	+	3.81	8.04	1	AGAAGAAG	WGAGGAAG	0.81	17	+<br>+<br>+	-501 -761<br>-174 -564<br>-868 -1449	260<br>390<br>581	97.9<br>211.4<br>351.8
☑	TNF	NM_000594	NF-kappaB	-	5.49	6.07	1	TGTGAATTCCCG	NGGGACTTTCCA	0.58	27	+<br>+	-501 -761<br>-174 -564	260<br>390	97.9<br>211.4
