# Supplementary material for: Sequence homology in eukaryotes (SHOE): interactive visual tool for promoter analysis
Source: BMC Genomics. 2018 Sep 27;19:715. doi: 10.1186/s12864-018-5101-3 (PMC6161448; doi:10.1186/s12864-018-5101-3)
Supplement: Supplementary file 3 — Figure S3. Visualization of ChiP-seq peaks from ArrayExperss database idenyified in SHOE predictions. A) Demosntrates two genes from overexpressed in mouse liver in Diabet 2 condition; B) Demonstrate TNF genes of mTOR human pathway in which promoter two peaks according ChIP-seq analysis have been identified. (ZIP 499 kb) [file 12864_2018_5101_MOESM3_ESM.zip › Supplementary Figure 3b.pdf]

mTOR pathway : TNF gene

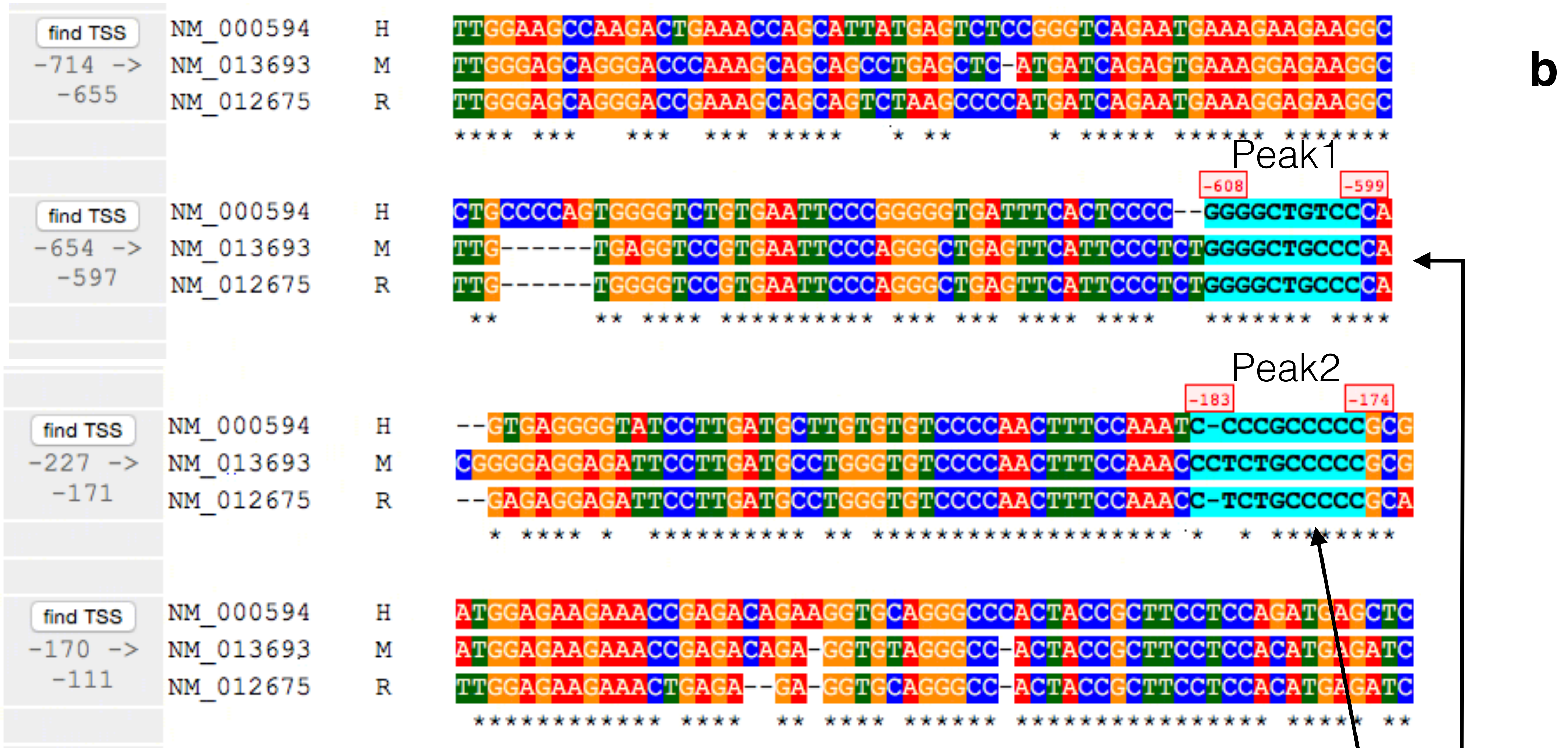

	gene	Refseq	TF	strand	MA score	PSSM score		MOTIF	CONSENSUS	SIM	Pareto	human +/-	human dist	human width	human score
☑	TNF	NM_000594	c-Rel	+	5.39	6.62	1	GGGGCTGTCC	SGGRNTTTCC	0.7	19	+<br>+<br>+	-501 -761<br>-174 -564<br>-868 -1449	260<br>390<br>581	97.9<br>211.4<br>351.8
☑	TNF	NM_000594	Sp-1	+	5.68	8.05	1	CCCCGCCCTC	CCCCGCCCCN	0.8	1	+<br>+<br>+	-501 -761<br>-174 -564<br>-868 -1449	260<br>390<br>581	97.9<br>211.4<br>351.8
☑	TNF	NM_000594	Sp-1	+	4.88	8.64	1	C-CCCGCCCCC	CCCCGCCCCN	0.55	1	+<br>+<br>+	-501 -761<br>-174 -564<br>-868 -1449	260<br>390<br>581	97.9<br>211.4<br>351.8
☑	TNF	NM_000594	STAT3	+	8.97	0.69	1	TGTGAATTCCCGGGGGTGATT	NGNNATTTCCSGGAARTGNNN	0.52	13	+<br>+<br>+	-501 -761<br>-174 -564<br>-868 -1449	260<br>390<br>581	97.9<br>211.4<br>351.8
☑	TNF	NM_000594	STAT1	+	3.82	8.05	1	GAATTCCC	CANTTCCS	0.69	15	+<br>+<br>+	-501 -761<br>-174 -564<br>-868 -1449	260<br>390<br>581	97.9<br>211.4<br>351.8
☑	TNF	NM_000594	PU.1	+	3.81	8.04	1	AGAAGAAG	WGAGGAAG	0.81	17	+<br>+<br>+	-501 -761<br>-174 -564<br>-868 -1449	260<br>390<br>581	97.9<br>211.4<br>351.8
☑	TNF	NM_000594	NF-kappaB	-	5.49	6.07	1	TGTGAATTCCCG	NGGGACTTTCCA	0.58	27	+<br>+	-501 -761<br>-174 -564	260<br>390	97.9<br>211.4
