# Supplementary material for: Sequence homology in eukaryotes (SHOE): interactive visual tool for promoter analysis
Source: BMC Genomics. 2018 Sep 27;19:715. doi: 10.1186/s12864-018-5101-3 (PMC6161448; doi:10.1186/s12864-018-5101-3)
Supplement: Supplementary file 1 — Figure S1. Pareto front score optimization on SHOE. A) Illustration of the conflict between two scores (MA score and PSSM score) and the concept of dominance; B) Visualization of several groups of scores using Pentachlorophenol response dataset (PCP), PCP_raw (red) denotes random score zone, PCP_ma (purple) denotes zone where MA score is high, PCP_pssm (blue) is the zone where the PSSM score is high, and PCPtrimmedLog (green) is the zone where a trade-off between two scores is taken. C) Example of the analysis with 30 top Pareto fronts with each type of motif shown in different shape and color. (PDF 360 kb) [file 12864_2018_5101_MOESM1_ESM.pdf]

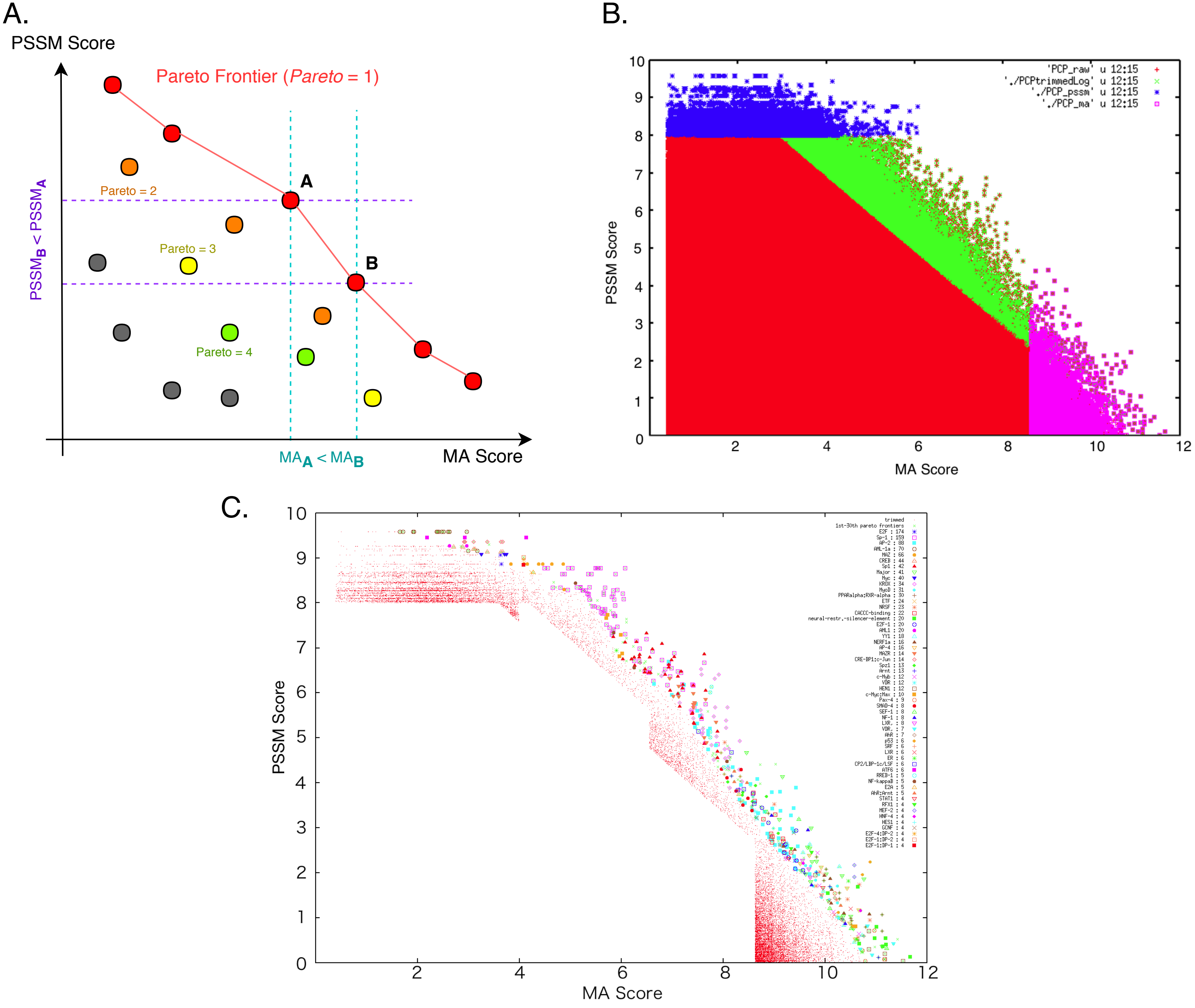
A.
PSSM Score
Pareto Frontier (Pareto = 1)
A
B
Pareto = 2
Pareto = 3
Pareto = 4
PSSM_B < PSSM_A
MA_A < MA_B
MA Score
B.
'PCP_raw' u 12:15
'./PCPtrimmedLog' u 12:15
'./PCP_pssm' u 12:15
'./PCP_ma' u 12:15
PSSM Score
MA Score
C.
trimmed
1st-30th pareto frontiers
PSSM Score
MA Score
